# Whole transcriptome analysis of chemically induced hepatocellular carcinoma using RNA‐sequencing analysis

**DOI:** 10.1002/2211-5463.12724

**Published:** 2019-09-29

**Authors:** Jie Sun, Jing Wang, Na Zhang, Renjun Yang, Keyang Chen, Derun Kong

**Affiliations:** ^1^ Department of Gastroenterology First Affiliated Hospital of Anhui Medical University Hefei China; ^2^ Department of Hygiene Inspection and Quarantine School of Public Health Anhui Medical University Hefei China

**Keywords:** dimethylnitrosamine, hepatocellular carcinoma, RNA‐sequencing, tetrachloride‐induced, transcriptome analysis

## Abstract

RNA‐sequencing (RNA‐Seq) is a useful method to explore the molecular events in cells and tissues at the transcriptional level. However, comprehensive transcriptome analysis of hepatocarcinogenesis and progression is lacking. In this study, we aimed to characterize a dimethylnitrosamine (DEN) and carbon tetrachloride (CCl_4_; DEN+CCl_4_)‐induced hepatocellular carcinoma (HCC) mouse model by RNA‐Seq. In total, 2033 genes were up‐regulated and 841 genes were down‐regulated after DEN and CCl_4_ stimulation. The differentially expressed genes were highly enriched for the Gene Ontology terms oxoacid metabolic process, carboxylic acid metabolic process, and organic acid metabolic process. Kyoto Encyclopedia of Genes and Genomes pathway analysis revealed that the top five significantly overrepresented pathways were metabolic pathways, chemical carcinogenesis, steroid hormone biosynthesis, retinol metabolism and metabolism of xenobiotics by cytochrome P450. Moreover, a protein‐protein interaction network analysis indicated that Rous sarcoma oncogene (Src) may play a key role in DEN+CCl_4_‐induced HCC. These results provide a comprehensive overview of transcriptome events in DEN+CCl_4_‐induced HCC.

**Abbreviations**
DEG: differentially expressed gene
CCl_4_: carbon tetrachloride
DEN: dimethylnitrosamine
GO: Gene Ontology
HCC: hepatocellular carcinoma
IP: intraperitoneally
KEGG: Kyoto Encyclopedia of Genes and Genomes
RNA‐Seq: RNA‐sequencing
Src: Rous sarcoma oncogene

Liver cancer is the fourth major cause of cancer deaths worldwide. In China, liver cancer is one of the five most commonly diagnosed cancers among males and tops the five leading causes of cancer deaths among males and females 1. Furthermore, hepatocellular carcinoma (HCC) is the main type of liver cancer 2. The refractory trait, complicated underlying mechanisms and individual differences in HCC are three stumbling blocks to the diagnosis and treatment of HCC 1.

Previous research has established chemically induced hepatocarcinogenesis rodent models with a multitude of similarities to human HCC for studies of the underlying mechanisms 3, 4.

However, whole transcriptome analysis of the dimethylnitrosamine (DEN) and carbon tetrachloride (CCl_4_; DEN+CCl_4_)‐induced HCC mice model is lacking. This dissertation aims to unravel the molecular mechanisms of underlying DEN+CCl_4_‐induced HCC at the transcriptional level using an RNA‐sequencing (RNA‐Seq) approach.

In brief, 2033 genes were up‐regulated and 841 genes were down‐regulated after DEN+CCl_4_ stimulation, as identified by RNA‐Seq. ‘Gene Ontology’ (GO) and ‘Kyoto Encyclopedia of Genes and Genomes’ (KEGG) pathway analysis showed that the genes were enriched for the GO terms – oxoacid metabolic process, carboxylic acid metabolic process, and organic acid metabolic process in both the high‐ and low‐dose groups – as well as the key pathways – metabolic pathways, chemical carcinogenesis, steroid hormone biosynthesis, retinol metabolism and metabolism of xenobiotics by cytochrome P450. A protein‐protein interaction network analysis indicated that Rous sarcoma oncogene (Src) may play a key role in DEN+CCl_4_‐induced HCC.

## Materials and methods

### Animals, treatments and tissue collection

C57BL/6J mice were purchased from the Model Animal Research Center (Nanjing University, Nanjing, China). All animals were housed in animal facilities at the Anhui Medical University under a standard 12‐h light/dark cycle with access to chow and water *ad libitum*. Experiments were conducted in accordance with the ethical statement of the Institutional Animal Care and Use Committee of Anhui Medical University.

Fourteen‐day‐old male C57BL/6J mice were treated with DEN intraperitoneally (IP; N0258‐1G; Sigma, St. Louis, MO, USA) at a dose of 25 mg·kg^−1^ (low‐dose group) or 40 mg·kg^−1^ (high‐dose group), or with vehicle alone (normal saline, IP, referred as control). Four weeks after their birth, the mice of the low‐dose group were treated with CCl_4_ (IP; SINOPHARM, Beijing, China) at 2.5 μL·g^−1^ once a week for a total of 25 doses. Eight weeks after their birth, the mice of the high‐dose group received 5 μL·g^−1^ CCl_4_ treatment twice a week for a total of 39 doses. CCl_4_ was diluted 1 : 4 in corn oil (C8267; Sigma). The solution was filtered through 0.22‐μm filter (Merck Millipore, Billerica, MA, USA). The mice of the control group were treated with corn oil alone. Finally, mice were sacrificed, and livers and serum samples were collected. Liver and body weights of mice were noted at the time of sacrifice and used to determine liver/body weight ratios.

### Histology and hematoxylin and eosin staining

Mouse liver tissues were fixed in 4% paraformaldehyde, embedded in paraffin and sectioned. Liver sections (5 μm) were stained with hematoxylin and eosin staining reagents. Images were captured using the Leica microscope (Wetzlar, Germany).

### RNA extraction and whole transcriptome sequencing

Total RNA was extracted with TRIzol (Invitrogen, Carlsbad, CA, USA) according to the manufacturer's protocol. The quality of total RNA was assessed by the Agilent Bioanalyzer 2100 (Santa Clara, CA, USA). Equal quantities of RNA from three samples in one group were mixed. The mRNA was purified and fragmented. Double‐stranded cDNA was synthesized and purified before adaptors were ligated to the fragments. PCR enrichment, library quality control and cluster generation were performed. Finally, the library products were sequenced by synthesis via Illumina HiSeq 2000 (San Diego, CA, USA).

### Analysis of RNA‐Seq data

Because some adaptor sequences and/or low‐quality reads are present in the raw reads, data filtering is carried out to obtain high‐quality reads as the clean reads (clean data). Clean reads were mapped to reference sequences and/or the reference gene set using SOAP aligner/SOAP2 (Short Oligonuclotide Analysis package). No more than two mismatches were allowed in the alignment. Then, a comprehensive bioinformatics analysis was used to enrich the dataset for genes that were most likely to be associated with the development of DEN+CCl_4_‐induced HCC, including clustering analysis, GO analysis, pathway analysis and dynamic gene network analysis.

### Statistical analysis

Data are expressed as means ± standard error of the mean. Differences between groups were tested for statistical significance using Student's *t*‐tests or one‐way ANOVA followed by Newman‐Keuls multiple comparison tests using graphpad prism 7.0 (La Jolla, CA, USA). All analyses with *P*‐value < 0.05 were considered significant.

## Results

### DEN and CCl_4_ stimulated HCC in mice

**Figure 1:**
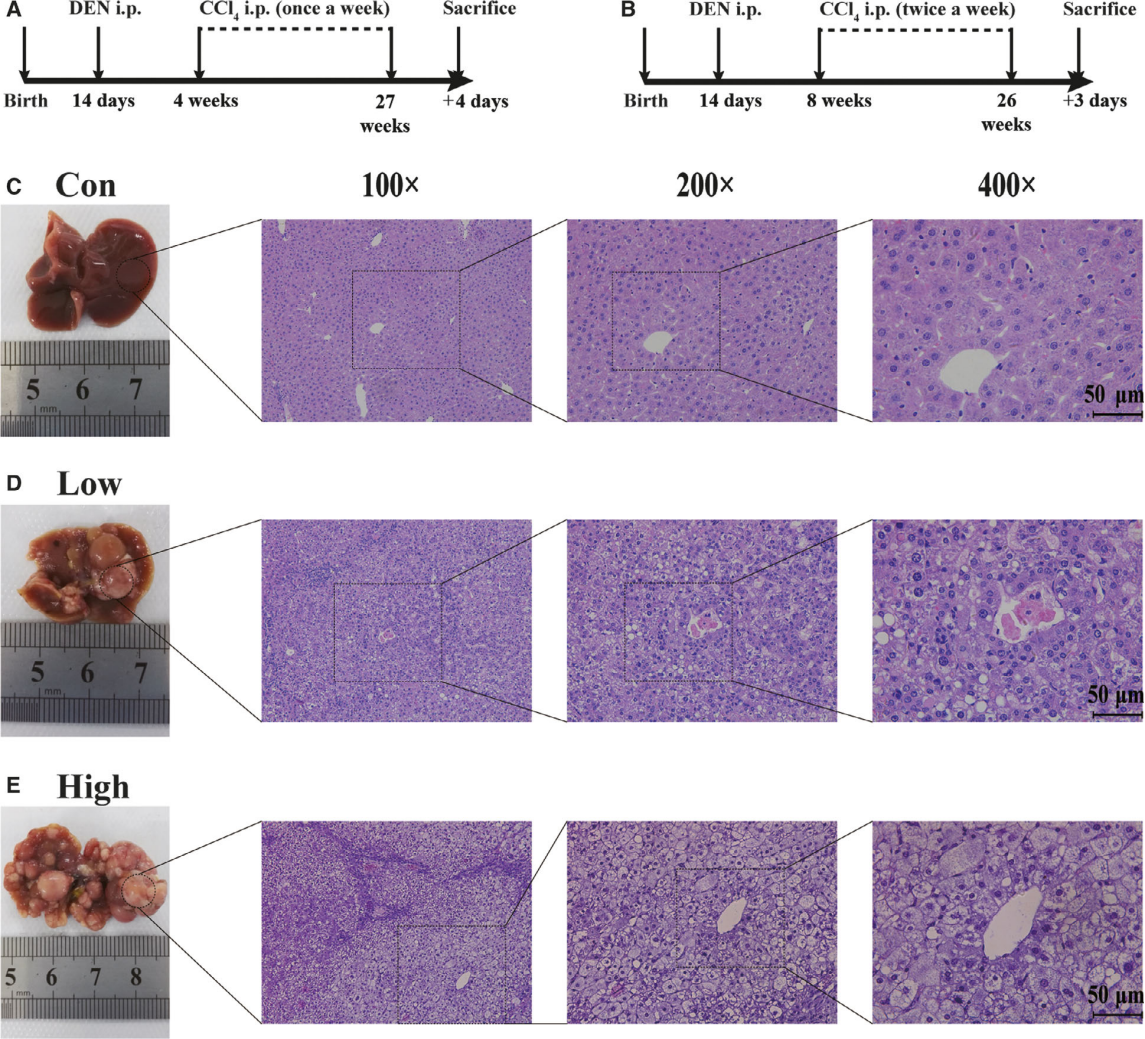
DEN+CCl_4_‐stimulated HCC in mice. (A, B) Schematic depiction of the experimental design of the HCC mouse model induced by DEN and CCl_4_. (C–E) Tumor and liver samples were identified macroscopically and microscopically. Hematoxylin and eosin staining of liver sections from treated and control mice (original magnification from left to right: ×100, ×200, and ×400; scale bar: 50 μm). Con, control; i.p., intraperitoneally.

In the HCC mice model, treatment with both DEN and CCl_4_ effectively induced liver tumors (Fig. 1A,B). Each mouse developed a liver tumor; however, a significantly higher number of tumors were detected in the high‐dose group than in the low‐dose group, and the tumor cells exhibited high‐grade atypia (Fig. 1C–E). The observed phenotypes indicated that DEN and CCl_4_ stimulated HCC successfully.

### Identification of differentially expressed genes

**Figure 2:**
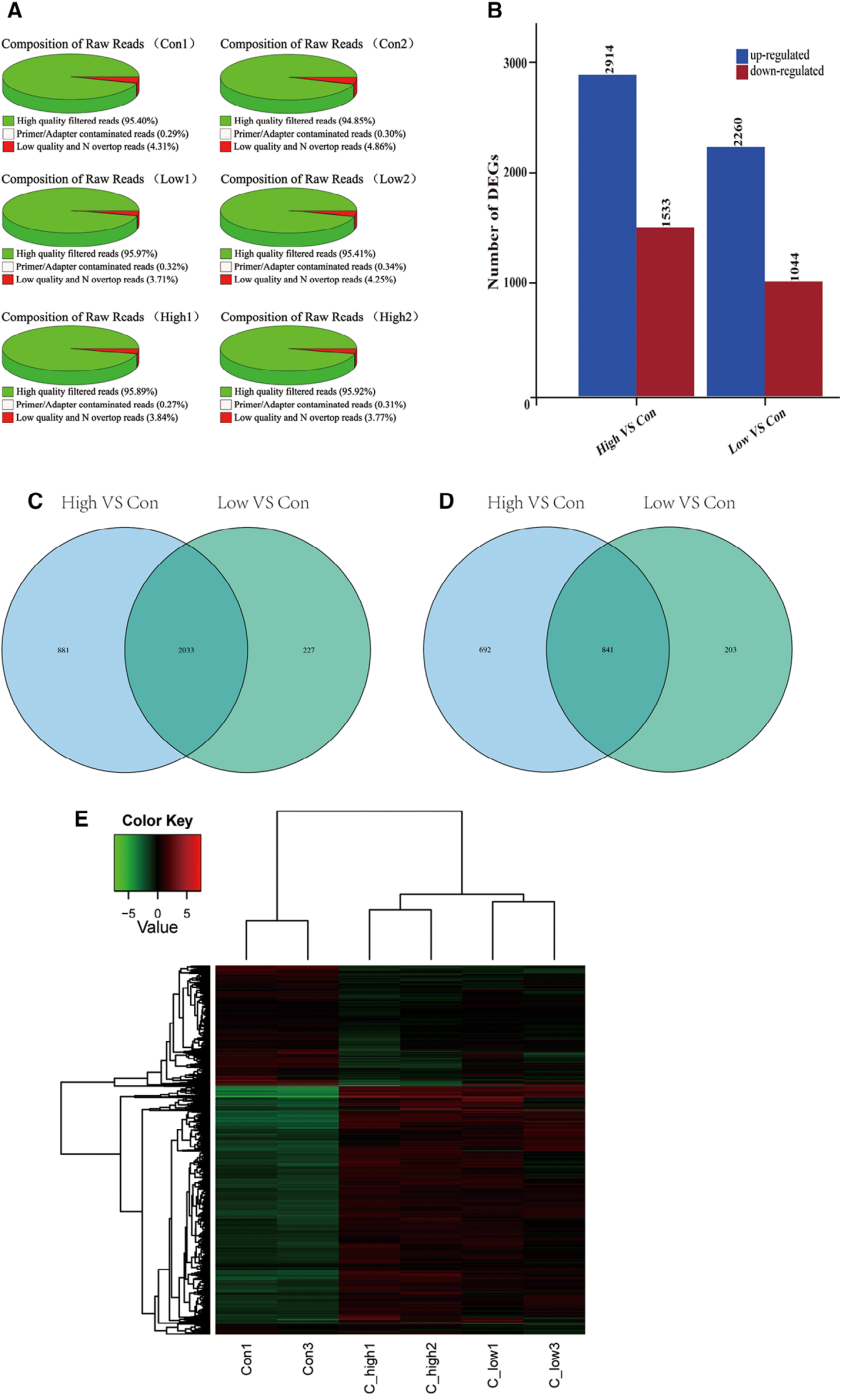
Distributions of up‐regulated and down‐regulated genes. (A) Composition of raw reads after DEN and CCl_4_ injection. (B) Bar graph shows the number of up‐regulated and down‐regulated genes in the low‐ and high‐dose groups compared with the control group. (C) Venn diagram of up‐regulated genes in both the high‐ and low‐dose groups. (D) Venn diagram of down‐regulated genes in both the high‐ and low‐dose groups. *P* ≤ 0.05; |Log2FC| ≥ 1. (E) Heatmap of DEGs among the three groups. Con, control.

To investigate differentially expressed genes (DEGs) in the DEN+CCl_4_‐induced HCC mouse model, we performed RNA‐Seq. As shown in Fig. 2A, after filtering primer‐ or adaptor‐contaminated and low‐quality reads, clean reads exceeded 94.8% of raw reads in six samples.

To identify DEGs, we used *P* ≤ 0.05 and |Log2FC| ≥ 1 as the threshold. When compared with genes in the control group, 2914 genes were significantly up‐regulated and 1533 genes were down‐regulated in the high‐dose group, and 2260 genes were markedly up‐regulated and 1044 genes were down‐regulated in the low‐dose group (Fig. 2B). Moreover, 2033 genes were up‐regulated in both the high‐dose and low‐dose groups when compared with levels in the control group (Fig. 2C). In addition, 841 genes were down‐regulated in both the high‐dose and low‐dose groups when compared with levels in the control group (Fig. 2D).

To investigate the functional correlation of genes with similar expression patterns, we performed a cluster analysis of expression patterns of DEGs (Fig. 2E). Expression differences are shown in different colors; green represents relatively low expression, and red represents relatively high expression.

### GO analysis

**Figure 3:**
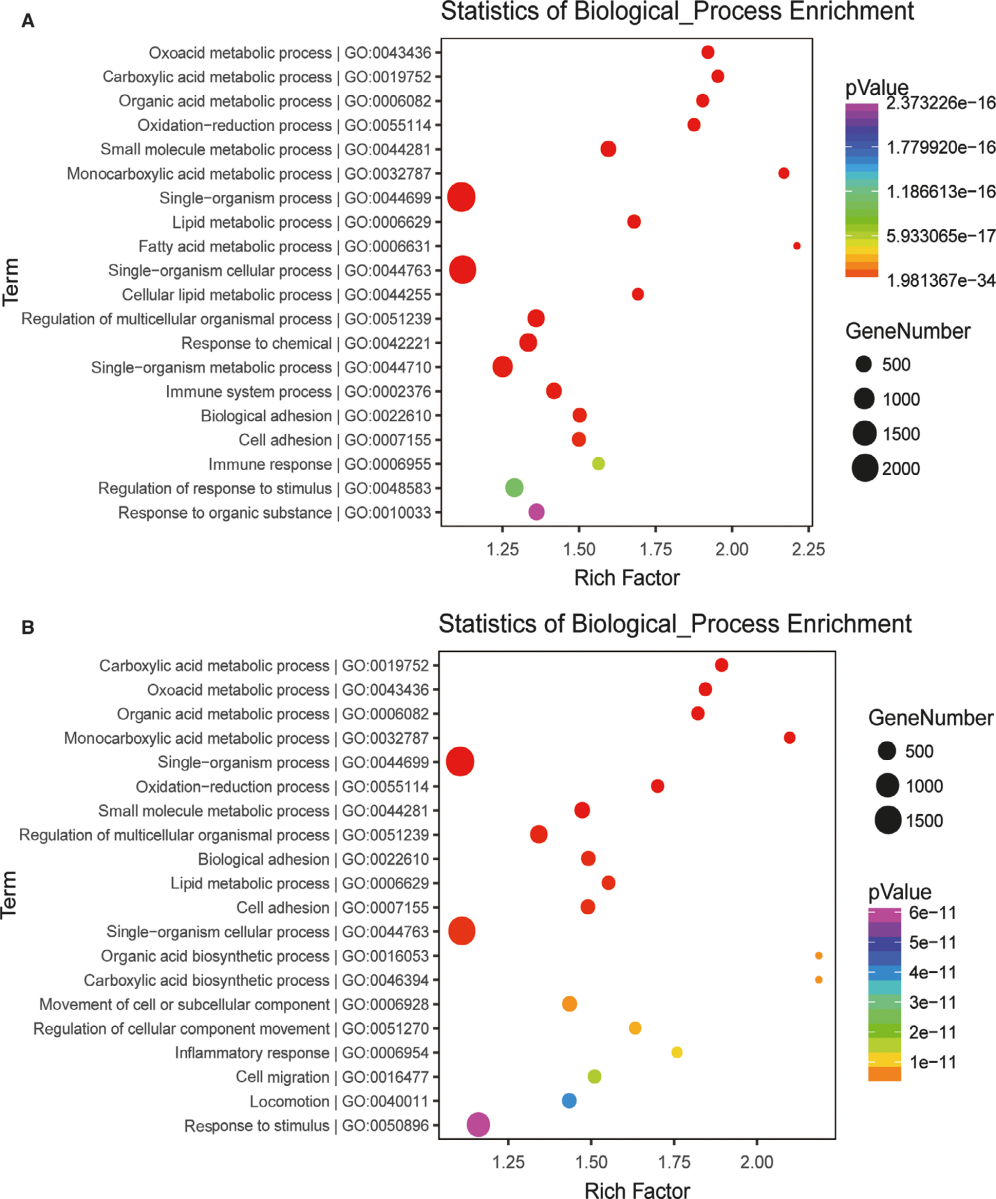
GO analysis. Significant GO terms for DEGs between the high‐dose group (A) or the low‐dose group (B) and the control group.

The GO tool was used for the functional classification of DEGs. Figure 3 summarizes the most significantly overrepresented GO terms in the biological process category. When compared with the control group, DEGs in the high‐dose group were most highly enriched for the GO terms oxoacid metabolic process, carboxylic acid metabolic process, organic acid metabolic process, oxidation‐reduction process and small molecule metabolic process; DEGs in the low‐dose group were enriched for carboxylic acid metabolic process, oxoacid metabolic process, organic acid metabolic process, monocarboxylic acid metabolic process and single‐organism process. The processes identified in this analysis are likely to contribute to the pathobiology of DEN+CCl_4_‐stimulated HCC.

### KEGG pathway analysis of DEGs

**Figure 4:**
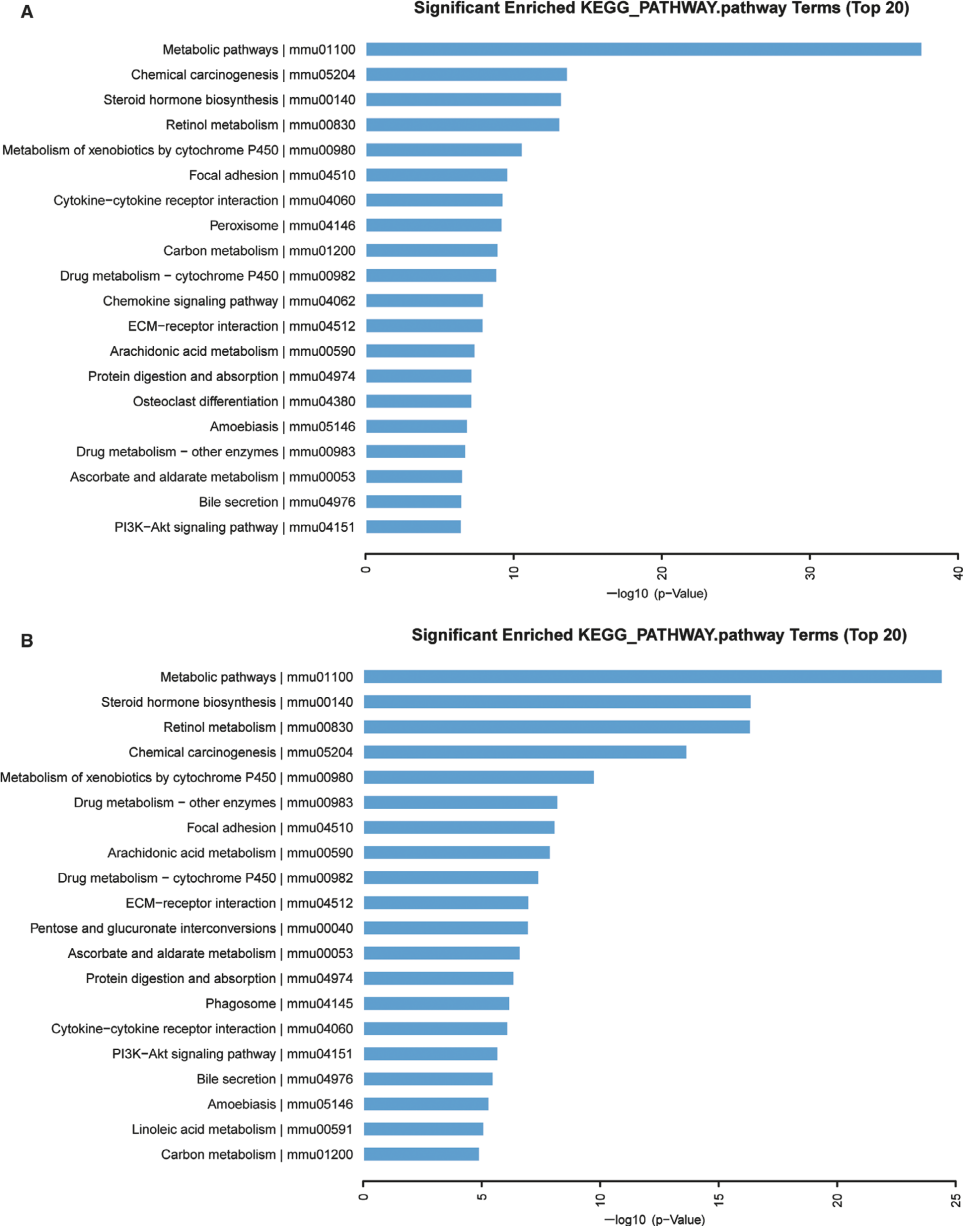
Kyoto Encyclopedia of Genes and Genomes pathway enrichment analysis. Significant pathways for DEGs between the high‐dose group (A) or the low‐dose group (B) and the control group.

To further understand the biological functions of genes, we performed a KEGG pathway enrichment analysis. The top five significantly overrepresented pathways in the high‐dose group were metabolic pathways, chemical carcinogenesis, steroid hormone biosynthesis, retinol metabolism and metabolism of xenobiotics by cytochrome P450, and in the low‐dose group were metabolic pathways, steroid hormone biosynthesis, retinol metabolism, chemical carcinogenesis and metabolism of xenobiotics by cytochrome P450 (Fig. 4). These pathways could be important for DEN+CCl_4_‐stimulated HCC.

### Protein‐protein interaction network analysis

**Figure 5:**
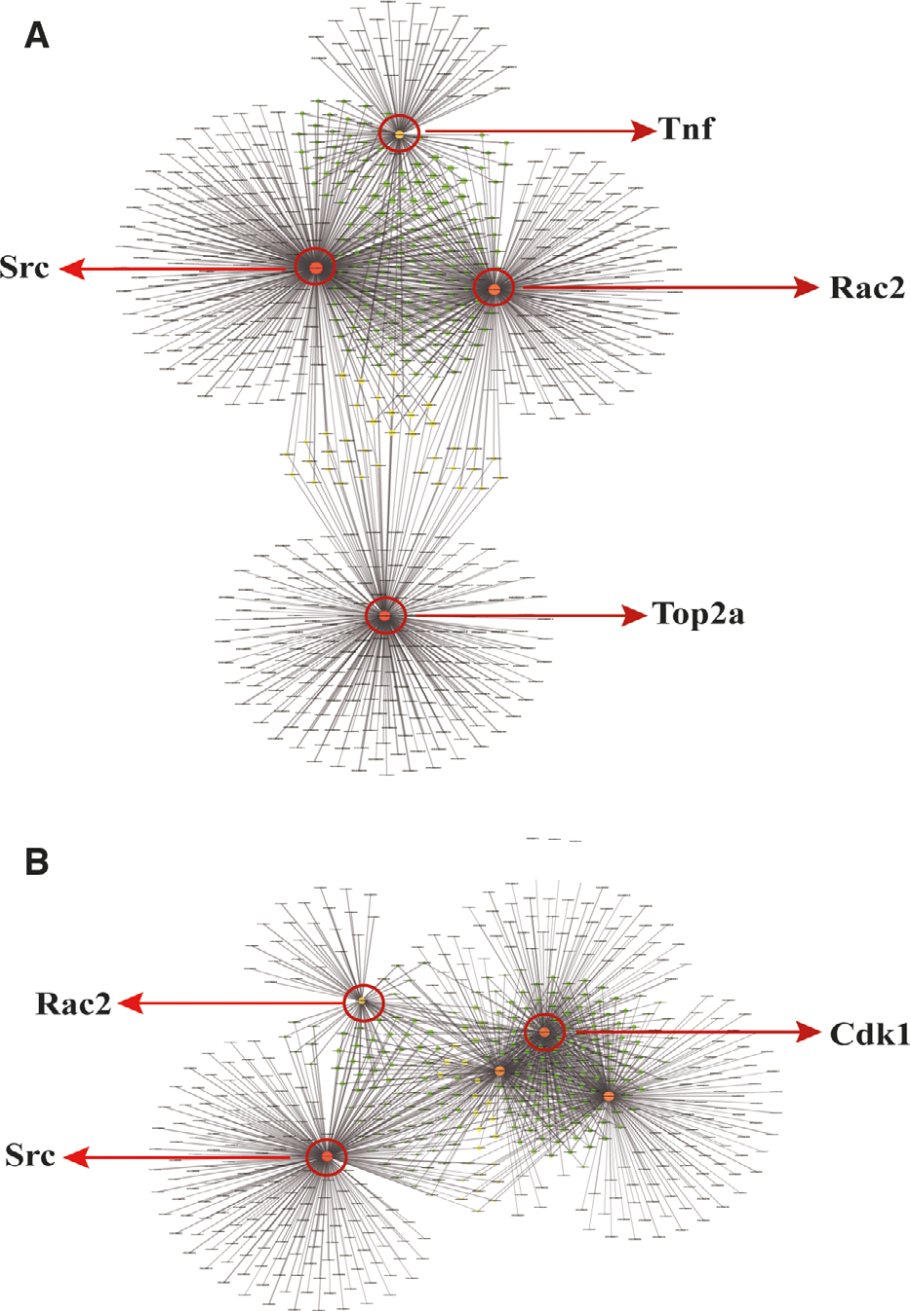
Protein‐protein interaction network analysis. (A) Network analysis of the DEGs in the high‐dose group versus the control group. (B) Network analysis of the DEGs in the low‐dose group versus the control group. Cdk1, cyclin‐dependent kinase 1; Rac2, Rac family small GTPase 2; Tnf, tumor necrosis factor; Top2a, topoisomerase (DNA) II alpha.

To investigate the important role of protein interactions in the DEN+CCl_4_‐stimulated HCC, we performed a protein‐protein interaction network analysis using the STRING online database (https://string‐db.org) and cytoscape. Figure 5A summarizes the correlation results for 759 DEGs between the high‐dose group and the control group. Tumor necrosis factor interacted with 108 proteins, Src connected with 363 proteins, Rac family small GTPase 2 had connections with 282 proteins and topoisomerase (DNA) II alpha had connections with 250 proteins. Figure 5B summarizes the results for 576 DEGs between the low‐dose group and the control group. In this analysis, Rac family small GTPase 2 interacted with 72 proteins, cyclin‐dependent kinase 1 interacted with 225 proteins and Src interacted with 363 proteins. Taken together, Src interacted with the most genes mostly in both the high‐ and low‐dose groups, suggesting that it may play a paramount role in DEN+CCl_4_‐stimulated HCC.

## Discussion

The current study investigated the gene expression characteristics, and mechanisms underlying HCC stimulated by DEN and CCl_4_ were evaluated by RNA‐Seq. A total of 2033 genes were up‐regulated and 841 genes were down‐regulated after DEN and CCl_4_ stimulation. These genes were overrepresented for the GO terms oxoacid metabolic process, carboxylic acid metabolic process, and organic acid metabolic process in both the high‐ and low‐dose groups, and for the KEGG pathways metabolic pathways, chemical carcinogenesis, steroid hormone biosynthesis, retinol metabolism, and metabolism of xenobiotics by cytochrome P450 in both the high‐ and low‐dose groups. These functions and pathways may be important in the pathological process of HCC induced by DEN and CCl_4_. Furthermore, the results of a gene network analysis indicated that Src may play a huge role in the regulation of DEN+CCl_4_‐induced HCC.

It is well acknowledged that most patients with HCC frequently exhibit cirrhosis 5. The mouse model induced by DEN and CCl_4_ can mimic the development from cirrhosis to HCC, similar to human HCC 4. It is therefore helpful to illustrate the pathophysiology and molecular and cellular mechanisms underlying HCC. Although some protocols have suggested that tumors might develop in C3H and B6C3F1/J mice earlier than in C57BL/6 mice 6, tumors developed successfully in all C57BL/6 mice in our study. Moreover, the C57BL/6 strain is more affordable and accessible.

Data were collected by RNA‐Seq. The advantages of this approach are as follows: first, transcriptome analysis offers complete information of tissues or cells at a particular period or time point 7. Second, RNA‐Seq can detect an undiscovered genome sequence with a low background signal and requires fewer RNA samples compared with microarrays 8. Third, RNA‐Seq has a better accuracy and repeatability 9, 10, 11, 12. Advances in RNA‐Seq have enabled the examination of HCC at high resolution 13, 14, 15, 16, 17.

Alterations in HCC at the transcriptional level reflect its histological and molecular heterogeneity 5. Transcriptome analysis can provide an overview of gene expression in the physiological state, but alternative splicing, microRNAs, long noncoding RNAs, and hypomethylation or hypermethylation are involved in carcinogenesis, progression and survival in HCC 13, 14, 15, 16. Future studies on the current topic are suggested to develop a full picture of the mechanism underlying HCC.

In general, we performed a whole transcriptome analysis of the DEN+CCl_4_‐induced HCC model by RNA‐Seq analysis. The findings of this research provide insights into the pathogenesis and molecular mechanism underlying HCC.

## Conflict of interest

The authors declare no conflict of interest.

## Author contributions

JS performed the majority of experiments, analyzed the data and wrote the paper. JW performed the molecular investigations and the treatment of animals. NZ and RY participated equally in the molecular investigations and the treatment of animals. KC provided the experimental equipment. DK designed and coordinated the research, as well as the critical revision of the manuscript.
